# Dose–volume histogram parameters of high-dose-rate brachytherapy for Stage I–II cervical cancer (≤4cm) arising from a small-sized uterus treated with a point A dose-reduced plan

**DOI:** 10.1093/jrr/rru006

**Published:** 2014-02-23

**Authors:** Akiko Nakagawa, Tatsuya Ohno, Shin-ei Noda, Nobuteru Kubo, Keiko Kuwako, Jun-ichi Saitoh, Takashi Nakano

**Affiliations:** 1 Department of Radiation Oncology, Gunma University Graduate School of Medicine, 3-39-22 Showa, Maebashi, 371-8511, Japan; 2 Gunma University Heavy Ion Medical Center, Gunma University, 3-39-22 Showa, Maebashi, 371-8511, Japan

**Keywords:** cervical cancer, CT-guided brachytherapy, small-sized uterus, late rectal complication, point A dose

## Abstract

We investigated the rectal dose-sparing effect and tumor control of a point A dose-reduced plan in patients with Stage I–II cervical cancer (≤4 cm) arising from a small-sized uterus. Between October 2008 and August 2011, 19 patients with Stage I–II cervical cancer (≤4 cm) were treated with external beam radiotherapy (EBRT) for the pelvis and CT-guided brachytherapy. Seven patients were treated with brachytherapy with standard loading of source-dwell positions and a fraction dose of 6 Gy at point A (conventional brachy-plan). The other 12 patients with a small uterus close to the rectum or small intestine were treated with brachytherapy with a point A dose-reduction to match D2cc of the rectum and <6 Gy as the dose constraint (‘point A dose-reduced plan’) instead of the 6-Gy plan at point A (‘tentative 6-Gy plan’). The total doses from EBRT and brachytherapy were added up and normalized to a biological equivalent dose of 2 Gy per fraction (EQD2). The median doses to the high-risk clinical target volume (HR-CTV) D90 in the conventional brachy-plan, tentative 6-Gy plan and point A dose-reduced plan were 62 GyEQD2, 80 GyEQD2 and 64 GyEQD2, respectively. The median doses of rectal D2cc in the corresponding three plans were 42 GyEQD2, 62 GyEQD2 and 51 GyEQD2, respectively. With a median follow-up period of 35 months, three patients developed Grade-1 late rectal complications and no patients developed local recurrence. Our preliminary results suggested that CT-guided brachytherapy using an individualized point A dose-reduced plan might be useful for reducing late rectal complications while maintaining primary tumor control.

## INTRODUCTION

Stage IB1–IIA2 cervical cancer can be cured by radiation therapy (RT) or surgery with similar effectiveness, but the rates and types of complications differ between the modalities [1]. With radical hysterectomy and pelvic lymphadenectomy, the most common adverse effects are lymphedema and urinary complications [2], whereas with RT the major sites of complications are the rectum, bladder and small bowel [3]. In particular, late rectal complication is one of the most important dose-related toxicities, occurring earlier than other late complications [4].

In Japan, the standardized treatment protocol for non-bulky (≤4 cm) Stage I and II cervical cancer is whole pelvic external beam radiation therapy (EBRT) of 20 Gy in 10 fractions, followed by pelvic EBRT with center shields of 30 Gy in 15 fractions and high-dose-rate intracavitary brachytherapy (HDR-ICBT) of 24 Gy in four fractions at point A [3]. A prospective multi-institutional study using this regimen in patients with non-bulky Stage I and II uterine cervical cancer showed a 2-year pelvic control rate of 96% [3]. The rates of late complications of Grade 1 or 2 were reported as 18% in the rectosigmoid colon, 0% in the bladder, and 4% in the small intestine [3].

Brachytherapy is an important component of RT for cervical cancer. Since point A is usually located outside the uterus in the case of a non-bulky tumor arising from a small-sized uterus, the conventional plan with 6 Gy at point A may result in overdosage for organs at risk (OARs) in close proximity. Orton *et al.* reported that increasing the dose of fractionation increases the risk of late complications in HDR-ICBT for cervical cancer [5]. In view of this, point A dose-reduced brachytherapy can be applied for selected patients with a non-bulky tumor, and individualized treatment planning with HDR-ICBT by use of computed tomography (CT)/magnetic resonance imaging (MRI) is beneficial for decreasing late complications while maintaining tumor control of the primary site.

Over the past 10 years, 3D image-guided brachytherapy (IGBT) has evolved. Based on treatment-planning studies, IGBT has the potential to optimize primary tumor dosimetry and to reduce the dose to OARs, particularly in patients with small tumors [6, 7, 8]. However, data supporting its clinical efficacy and safety are still insufficient.

In the present study, we compared dose–volume histogram parameters for the point A dose-reduced plan, the tentative 6-Gy plan (i.e. the original plan before reducing the point A dose), and the conventional-brachy plan without necessarily reduction of the point A dose. The purpose of the study was to evaluate whether brachytherapy by a point A dose-reduced plan is useful for decreasing the incidence of late rectal complications while retaining acceptable primary tumor control, especially in patients with a small-sized uterus.

## MATERIALS AND METHODS

### Patients

Between October 2008 and August 2011, 19 patients with histologically confirmed Stage I–II cervical cancer (**≤** 4 cm) underwent definitive RT at Gunma University Hospital. Written informed consent was obtained from all patients before treatment. Tumor stage was determined according to the FIGO classification. All patients underwent MRI scanning as part of the pretreatment evaluations, and maximum tumor size and uterine size (cranio–caudal length and anterior–posterior diameter of the cervix) were measured. Of the 19 patients, 7 patients were treated with EBRT for the pelvis and IGBT with a point A dose of 6 Gy using in-room CT (the ‘conventional brachy-plan group’). The median age was 57 years (range, 43–76 years). Five of these patients had Stage IB1 and two had Stage IIB cervical cancer. One patient had adenosquamous carcinoma and the others squamous cell carcinoma. The other 12 patients were treated with EBRT for the pelvis and IGBT with a point A dose-reduced plan (the ‘point A dose-reduced plan group’). In this group, the median age was 71 years (range, 54–80 years). Four of these patients had Stage IB1, five had Stage IIA1, and three had Stage IIB cervical cancer. Three patients had adenocarcinoma and the others squamous cell carcinoma.

### Radiotherapy

Patients were treated with a combination of EBRT and HDR-ICBT. EBRT was delivered with CT-based treatment planning, with a dose of 2 Gy per fraction, five times per week. As a standard, a midline block was inserted for a total dose of 20 Gy for squamous cell carcinoma, or 30 Gy for adenocarcinoma. Pelvic irradiation with a midline block 3 cm in width was performed with doses of 2 Gy per fraction to a total dose of 50 Gy. None of the patients received chemotherapy.

Along with the central shielding irradiation, IGBT was started with a remote after-loading system (micro-Selectron^®^, Nucletron, an Elekta Company, Veenendaal, The Netherlands) using HDR ^192^Ir sources. Four fractions of IGBT were carried out once a week for most cases. Based on the tumor response, the application of a fifth IGBT was an option for adenocarcinoma.

A set of Fletcher-Suit Asian Pacific applicators (tandem and half-size ovoid, Nucretron) was used. Applicators were implanted into the patient's vagina and uterus on the couch of the in-room CT, and all processes including application, imaging and irradiation could be performed on the same couch. After implantation, CT scans were generated on the same couch with 3-mm slice thickness. MRI scans were taken before the first brachytherapy for all patients and used as reference images of the tumor.

### Contouring and treatment planning

**Fig. 1.**
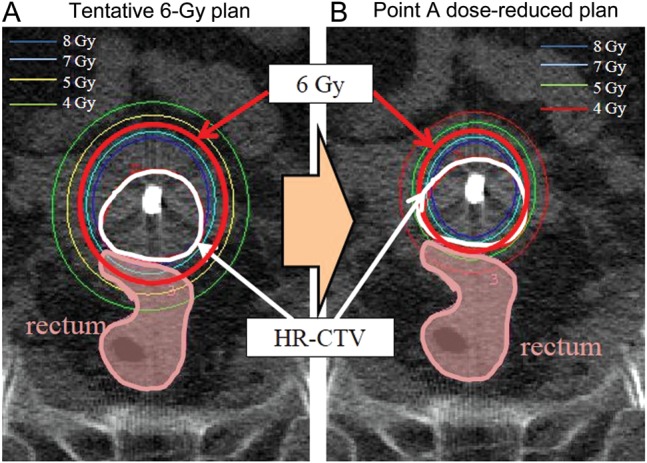
Dose distribution of brachytherapy. (**A**) Tentative 6-Gy plan. (**B**) Point A dose-reduced plan. Red line: 6-Gy line. White line: high risk clinical target volume (HR-CTV). Pink area: rectum.

CT-guided brachytherapy planning was performed in each brachytherapy session. Data were transferred from the CT scans into the PLATO^®^ treatment planning system (Nucletron) for contouring and planning. The high-risk clinical target volume (HR-CTV) and OARs were delineated following the Gynaecological Groupe Européen de Curiethérapie–European Society for Therapeutic Radiology and Oncology (GYN GEC-ESTRO) recommendations [9, 10]. The point A (traditional dose prescription point, defined as 20 mm superior to the external os and 20 mm lateral from the axis of the intrauterine tandem) was marked on corresponding slices of CT images as a reference starting point for dose optimization. After standard loading of source dwell positions and weighting in tandem and ovoid with a fraction dose of 6 Gy at point A in all patients, if the 6-Gy isodose line was located between the HR-CTV and the rectum, sigmoid or small intestine, brachytherapy with point A dose 6 Gy was carried out (conventional brachy-plan, 7 patients). On the other hand, if the 6-Gy isodose line sufficiently covered not only the HR-CTV but also the rectum, sigmoid or small intestine, mainly because the uterus was small-sized, the point A dose was manually reduced while maintaining at least 6 Gy for the HR-CTV D90 (point A dose-reduced plan, 12 patients). This optimization was based on visual inspection of the isodose lines. The plan before reducing the point A dose was called the tentative 6-Gy plan. Figure 1 shows the dose distribution of representative cases. The doses of the HR-CTV D90 and D2cc of the rectum were compared for the three plans (conventional brachy-plan, tentative 6-Gy plan and point A dose-reduced plan).

The total doses from EBRT and IGBT were summarized and normalized to a biological equivalent dose of 2 Gy per fraction (EQD2) using the linear quadratic model with α/β = 3 Gy for the rectum and α/β = 10 Gy for the HR-CTV.

### Follow-up

After treatment, patients were followed-up at 1–3-month intervals for the initial 2 years and at 3–6-month intervals for the subsequent 3 years. Disease status and toxicities were assessed by oral history, physical examinations, appropriate laboratory tests, CT and MRI. Toxicities were classified as late complications if they occurred >90 days after RT according to the Radiation Therapy Oncology Group/European Organization of Research and Treatment of Cancer (RTOG/EORTC) late radiation morbidity scoring scheme.

### Statistical analysis

The Mann–Whitney U test was used to compare the distribution of dosimetric parameters for the conventional brachy-plan, the tentative 6-Gy plan and the point A dose-reduced plan. A value of *P* < 0.05 was considered significant.

## RESULTS

### Uterus size

Comparisons of the patient characteristic between the two treatment plan groups are shown in Table 1. The cranio–caudal length of the uterus was significantly shorter in the point A dose-reduced plan group compared with the conventional brachy-plan group (*P* = 0.001). There was no significant difference in tumor size or anterior–posterior diameter of the cervix between the two groups.

**Table 1.** Patient characteristics

	Point A dose-reduced plan group (*n = 12)*	Conventional brachy-plan group (*n* = 7)	*P*-value
Cranio–caudal length of the uterus (cm), median (range)	5.4 (3.7–6.6)	7.1 (6.2–9.3)	<0.001
Anterior–posterior diameter of the cervix (cm), median (range)	2.4 (1.7–3.3)	2.6 (2.3–3.6)	0.17
Tumor size at diagnosis (cm), median (range)	3.4 (1.8–3.8)	3.2 (1.6–3.6)	0.550
HR-CTV volume at first brachytherapy (cm^3^), median (range)	19 (7–33)	28 (11–41)	<0.001
EBRT (WP/CS)			
30 Gy/20 Gy	4	1	
20 Gy/30 Gy	8	6	
Point A dose per fraction, median (range)	5 Gy (4–6 Gy)	6 Gy	<0.001
Disease control			
NER	11	7	
LR	0	0	
DOPD	1	0	
Late rectal complication			
Grade 0	11	5	
Grade 1	1	2	

NER = no evidence of recurrence, LR = local recurrence, DOPD = died of primary disease, EBRT = external beam radiation therapy, WP = whole-pelvic radiation, CS = central shielding radiation.

### Clinical outcome

The median follow-up duration for all 19 patients was 35 months (range, 18–51 months), and 18 patients are alive without any recurrence. One patient treated with the point A dose-reduced plan developed liver metastases after radiation therapy. She died of primary disease 18 months after treatment.

In the conventional brachy-plan group, two of the seven patients developed a late rectal complication of Grade 1. Their total doses of D2cc of the rectum, based on the calculation of EBRT and ICBT, were 54 GyEQD2 and 42 GyEQD2, respectively.

In the point A dose-reduced plan group, only one patient developed a late rectal complication of Grade 1 at 7 months after treatment, but recovered at 11 months without any intervention. In this patient, the total dose of D2cc of the rectum based on the calculation of EBRT and ICBT was 54 GyEQD2.

### Comparison of dosimetric parameters

**Fig. 2.**
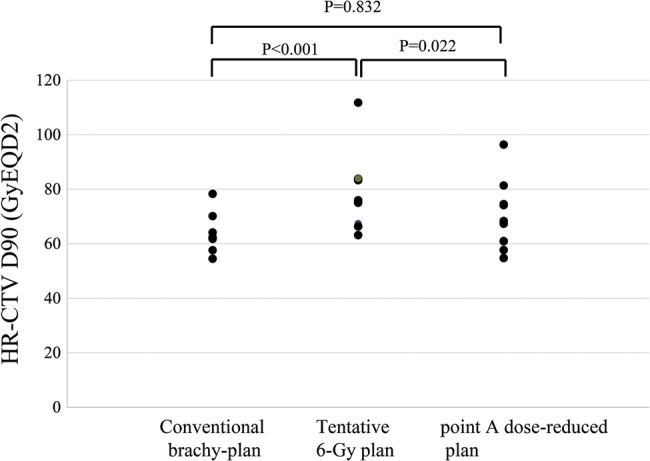
Comparison of HR-CTV D90 for the conventional brachy-plan, tentative 6-Gy plan and point A dose-reduced plan. The median doses to the HR-CTV D90 for the conventional brachy-plan, tentative 6 Gy plan and point A dose-reduced plan were 62 GyEQD2 (range, 54–78 GyEQD2), 80 GyEQD2 (range, 63–112 GyEQD2) and 64 GyEQD2 (range, 55–96 GyEQD2), respectively.

The HR-CTV at first brachytherapy was 28 cm^3^ for the conventional brachy-plan group and 19 cc for the point A dose-reduced plan group. The volume was significantly smaller in the point A dose-reduced plan group compared with that in the conventional brachy-plan group (*P* < 0.001). Figure 2 shows a comparison of the HR-CTV D90 for the three plans. The median doses of the HR-CTV D90 for the conventional brachy-plan, tentative 6-Gy plan and point A dose-reduced plan were 62 GyEQD2 (range, 54–78 GyEQD2), 80 GyEQD2 (range, 63–112 GyEQD2) and 64 GyEQD2 (range, 55–96 GyEQD2), respectively. The median doses with ICBT alone of the HR-CTV D90 for the conventional brachy-plan, tentative 6 Gy-plan and point A dose-reduced plan were 42 GyEQD2 (range, 34–58 GyEQD2), 54 GyEQD2 (range, 43–83 GyEQD2) and 43 GyEQD2 (range, 27–66 GyEQD2), respectively.

**Fig. 3.**
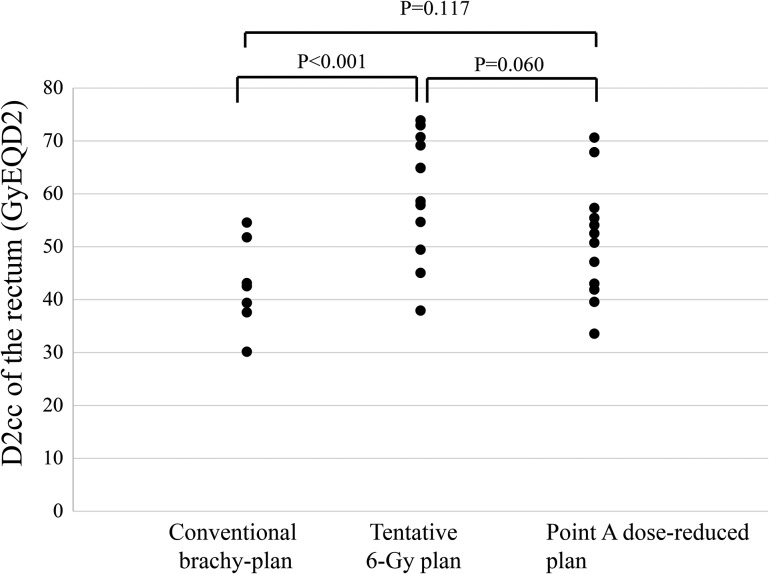
Comparison of D2cc of the rectum for the conventional brachy-plan, tentative 6-Gy plan and point A dose-reduced plan. The median dose to the D2cc of the rectum for the conventional brachy-plan, tentative 6 Gy plan and point A dose-reduced plan were 42 GyEQD2 (range, 30–54 GyEQD2), 61 GyEQD2 (range, 38–74 GyEQD2) and 51 GyEQD2 (range, 33–70 GyEQD2), respectively.

Figure 3 shows the comparison of D2cc of the rectum for the three plans. The median doses for D2cc of the rectum for the conventional brachy-plan, tentative 6-Gy plan and point A dose-reduced plan were 42 GyEQD2 (range, 30–54 GyEQD2), 62 GyEQD2 (range, 38–74 GyEQD2) and 51 GyEQD2 (range, 33–70 GyEQD2), respectively. Three patients demonstrated >70 GyEQD2 when the tentative 6-Gy plan was used. Two of these three patients were actually treated with the point A dose-reduced plan, in which D2cc of the rectum was reduced to <70 GyEQD2. The median dose with ICBT alone for D2cc of the rectum for the conventional brachy-plan, tentative 6-Gy plan and point A dose-reduced plan were 22 GyEQD2 (range, 10–32 GyEQD2), 37 GyEQD2 (range, 18–54 GyEQD2) and 25 GyEQD2 (range, 13–40 GyEQD2), respectively. In the tentative 6-Gy plan, D2cc of the rectum was >70 GyEQD2 in three patients.

## DISCUSSION

The present study was conducted to evaluate the potential benefit of dose adaptation at IGBT for non-bulky cervical cancer arising from a small-sized uterus. Treatment planning was done so as to reduce the rectal doses within acceptable levels while maintaining tumor control in patients with Stage I–II cervical cancer (≤4 cm) arising from a small-sized uterus treated with CT-based brachytherapy. However, dose constraints for rectal D2cc had not yet been standardized in the Japanese treatment schedule. Kato *et al.* evaluated the efficacy of CT-based dose–volume parameters of the rectum as a predictor of late rectal complications in 84 cervical cancer patients treated with RT alone [4]. RT was carried out according to the accepted treatment schedule in Japan. The dose–response relationship was observed between D2cc of the rectum and late rectal complications. Especially, the incidence of late rectal complications of Grade 1 or higher was significantly greater in patients receiving >70 GyEQD2. Isohashi *et al.* also demonstrated that a patient subgroup with both a high dose (over 71 GyEQD2) and a high source strength (over 2.4 cGy.m^2^.h^−1^) showed a significantly greater probability of late rectal bleeding of Grade 1 or higher [11]. Therefore, a dose–volume constraint <70 GyEQD2 for D2cc of the rectum may become a target for avoiding late complications with 3D-ICBT.

In the case of a non-bulky tumor (≤4 cm), a minimally required dose to the HR-CTV D90 for achieving excellent tumor control has yet to be established. Vienna University reported treatment planning aimed at a dose to the HR-CTV D90 of over 85 GyEQD2, and that a high dose to the HR-CTV was not significantly reduced, even for non-bulky and good-responding tumors, except when violating dose–volume constraints for OARs (70 GyEQD2 in D2cc for rectum and sigmoid colon, and 90 GyEQD2 in D2cc for bladder) [12]. We agree with their concept in principle but, compared with the dose they used, the D90 to the HR-CTV in our treatment was lower (median, 61 GyEQD2; range, 55–96 GyEQD2). Although no patients developed pelvic recurrence in our series, long-term observation will be necessary to confirm the efficacy.

In this report, we compared the HR-CTV D90 and D2cc to the rectum for three types of plans. In the point A dose-reduced plan, the HR-CTV D90 dose was lower than for that of the tentative 6-Gy plan. However, there was no significant difference in dose between the conventional brachy-plan and the point A dose-reduced plan. Similarly, there was no significant difference in the D2cc of the rectum between the conventional brachy-plan and the point A dose-reduced plan. This may indicate that the doses to the HR-CTV D90 and the D2cc of the rectum in the point A dose-reduced plan were comparable with our traditional prescription that has shown both efficacy and safety.

It is important to identify patients who will gain a benefit from the point A dose-reduced plan. The present study showed that the HR-CTV and the cranio–caudal length of the uterus were significantly different between the point A dose-reduced plan and conventional brachy-plan groups, although tumor size and anterior–posterior diameter of the cervix did not differ between the two groups. Zwahlen *et al*, indicated that IGBT was beneficial for a small HR-CTV (<16.1 cm^3^) [6], and our findings are in line with their conclusion. Even when the cervical tumor size was small at brachytherapy, the whole cervix was contoured as the HR-CTV. Therefore, in the case of a small tumor, the HR-CTV would be the more important factor for considering the point A dose-reduced plan when compared with tumor size. Additionally, the point A dose-reduced plan should be considered in balance with OARs, as when none of the rectum, sigmoid and small intestine is exposed to over 6 Gy, brachytherapy with the conventional brachy-plan is acceptable.

There is not yet sufficient information available on the clinical efficacy of the point A dose-reduced plan. In this study, we observed excellent pelvic control and acceptable incidence of late rectal complications. The incidence of late rectal complications of Grade 1 or higher was a reported 18% in 2D brachytherapy with 24 Gy in four fractions at point A [3]. In our study, in the point A dose-reduced plan group, only one patient (8%) developed late rectal complications of Grade 1. Due to the small number of patients and the short follow-up period, it is difficult to confirm whether or not our dose fractionation schedule and dose–volume concept are valid. With 3D-IGBT, it is a possible to deliver sufficient dose to the HR-CTV while keeping the dose to the rectum within acceptable levels. Further study will be needed to evaluate whether our approach achieves good balance between tumor control and late complications in patients with non-bulky cervical cancer arising from a small-sized uterus.
